# Associations of screen time and physical activity with suicidality in adolescents: a national cohort study

**DOI:** 10.1007/s00127-025-02827-1

**Published:** 2025-02-17

**Authors:** Rose Marie Wilkens Rasmussen, Katrine Strandberg-Larsen, Stine Danielsen, Merete Nordentoft, Annette Erlangsen, Trine Madsen

**Affiliations:** 1 Danish Research Institute of Suicide Prevention, Copenhagen Research Center for Mental Health, Mental Health Center Copenhagen, Copenhagen, Denmark; 2 Section of Epidemiology, Faculty of Health and Medical Sciences, University of Copenhagen, Copenhagen, Denmark; 3 Deptartment of Clinical Medicine, University of Copenhagen, Copenhagen, Denmark; 4 Department of Mental Health, Johns Hopkins Bloomberg School of Public Health, Baltimore, MD USA; 5 Center of Mental Health Research, College of Health and Medicine, The Australian National University, Canberra, Australia

**Keywords:** Suicidal ideation, Suicide attempt, Screen time, Physical activity, Adolescence, Epidemiology

## Abstract

**Purpose:**

Evidence linking screen time and physical activity with suicidality among adolescents is inconsistent. Our objective was to examine longitudinal associations between screen time and physical activity with suicidality among Danish adolescents.

**Methods:**

Self-reported data on daily screen time and physical activity at age 11 was obtained from 28 613 adolescents (60% females) who participated in the Danish National Birth Cohort and linked to information on suicidality at age 18, based on self-reports and register data. Adjusted relative risk ratios (aRRR) were estimated using multinomial logistic regressions, while accounting for parental socio-demographics, psychiatric history and child risk behaviours.

**Results:**

High levels of daily screen time (≥ 6 h) were reported by 6.9% and 12.6% of females and males, respectively. This was associated with suicidal ideation in females (aRRR:1.67, 95% CI:1.44–1.93) and suicide attempt in both sexes (females: aRRR:2.04 [1.51–2.75], males: aRRR:3.61 [1.89–6.89]) when compared to adolescents with < 2 h of daily screen time. Low levels of physical activity were reported by respectively 13.4% and 14.8% of females and males and was associated with increased risk of suicidal ideation (females: aRRR:2.18, [95% CI:1.94–2.44], males: aRRR:2.11 [1.83–2.43]) and suicide attempt (females: aRRR:2.27, [1.77–2.91], males: aRRR:2.49 [1.61–3.85]) when compared with those with the highest level. Independently of screen time level, males with low physical activity had higher risk for suicide attempt compared to males with a high level of physical activity.

**Conclusion:**

High levels of screen time and low levels of physical activity were associated with an increased risk of suicidality among adolescents.

**Supplementary Information:**

The online version contains supplementary material available at 10.1007/s00127-025-02827-1.

## Background

First onset of suicidality, defined as suicidal ideation and/or suicide attempt, often occurs during adolescence [1]. Suicidal ideation has been reported by 14.3-22.6% of individuals aged 6–21 years, while 4.6–15.8% stated having had a suicide attempt [2]. Suicide attempt is a strong predictor of later suicide [3], thus, identification of modifiable risk factors is imperative for designing suicide preventive interventions.

Existing evidence have linked higher levels of screen time to suicidality in adolescents aged 10–19 years [4–8]. Further, a dose-response association where a higher number of daily hours of screen time was linked to higher risks of suicidality has been reported [4, 7, 8]. Likewise, lower levels of physical activity in adolescents have been linked to higher risks of suicidality when compared to those with higher levels, although findings have been contradictory [5, 6, 8, 9]. In a systematic review and meta-analysis, seven out of fourteen studies showed an association between higher levels of physical activity and lower levels of suicidal ideation among adolescents, while the remaining seven studies found no association [9]. Nevertheless, high levels of physical activity have also been linked to elevated risks of suicidality, albeit mostly among adolescent females [10–12]. Interestingly, physical activity was assessed using comparable measures across these studies with diverging results. The current body of evidence is mainly based on cross-sectional data (only one study using longitudinal study [13] design was identified in literature search) making it difficult to separate whether high screen time and low physical activity might have led to suicidality or whether suicidality (i.e. low mental health) led to behaviors with higher screen time and lower physical activity. Further analyses have yet to account for relevant confounders, such as possible vulnerable factors in the child’s home environment like household income and parental history of mental disorders. Such factors may be hypothesized to impact a child’s time spent on screen and physical activity for instance through lower resources/mental energy in parents to setting screen time limits for the child and/or through lower income limiting possibilities for paying for child’s participation in team sports.

The aim of this study was to analyze whether self-reported levels of daily screen time and physical activity at age 11 were associated with suicidality until age 18.

## Methods

### Study design

We obtained longitudinal data from the Danish National Birth Cohort (DNBC) on 96 822 individuals born between 1996 and 2003. Multiple data collections have been conducted within the DNBC, including at age 11 (DNBC-11) during 2010–2014 and at age 18 years and three months (DNBC-18) during 2016–2021. In the DNBC-11 and the DNBC-18, data were collected from the adolescents via self-administered web-based questionnaires. Further details regarding DNBC are described elsewhere (www.dnbc.dk) [14]. Using the unique personal identification number assigned to all residents in Denmark [15], individual-level data on the DNBC participants and their parents were obtained from nationwide registers and linked to the survey data.

### Participants

**Fig. 1:**
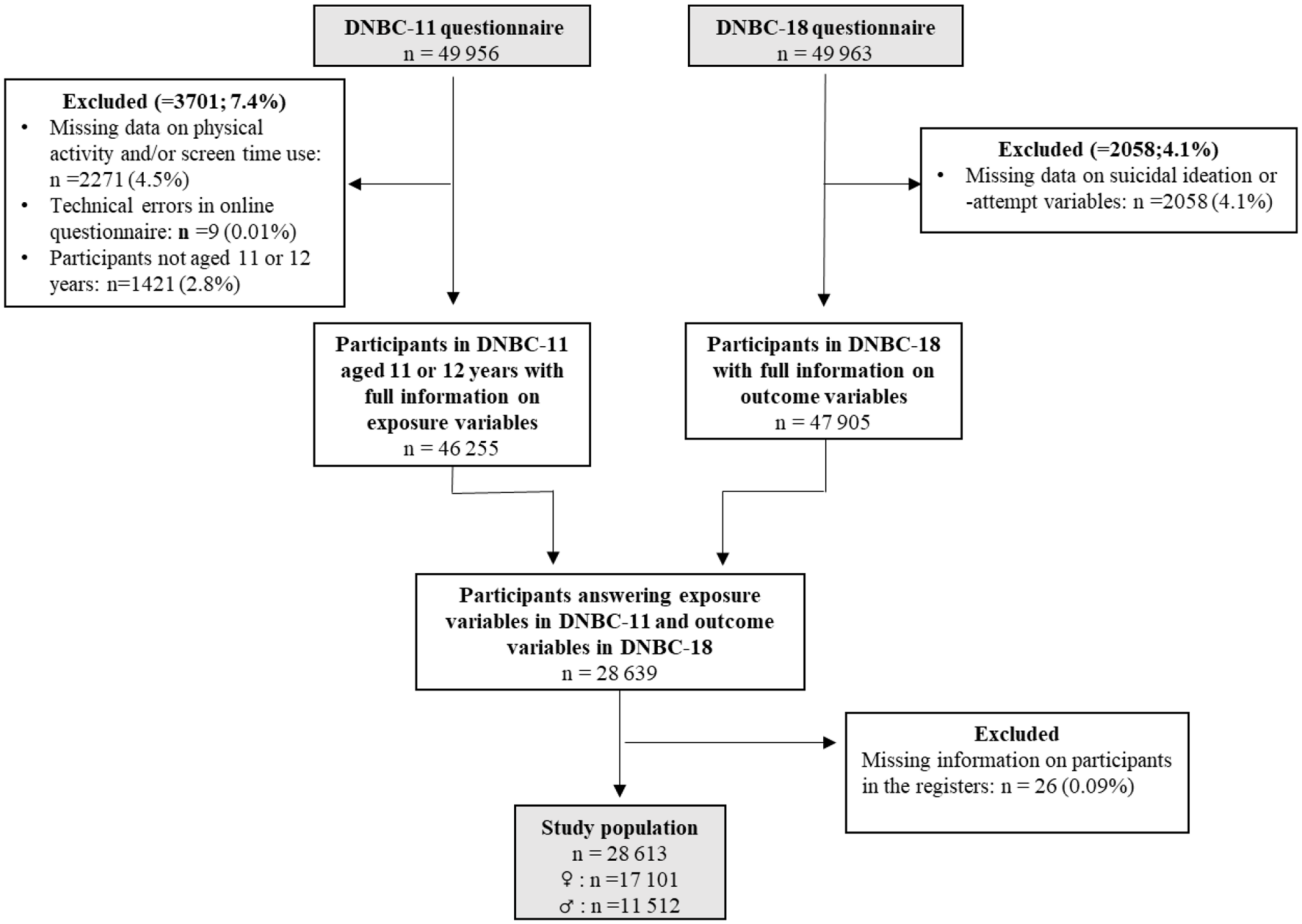
Flowchart of study population

DNBC participants were included if they had provided full information regarding exposures, i.e. screen time and physical activity, in DNBC-11 and outcome measures, i.e. suicidality, in DNBC-18 (Fig. 1). The sample consisted of 28 613 adolescents (17 101 females and 11 512 males).

### Screen time

At age 11, adolescents were asked how much of their leisure time they spend (1) in front of the computer and (2) watching TV/films (Supplementary Table 1). The number of screen time hours per day with respect to (1) and (2) were reported separately for weekdays and weekend days. The total number of screen time hours per day was calculated irrespective of type. Secondly, we combined the measures by assigning weights to the estimates of weekdays (5/7) and weekend days (2/7) and calculated the total average of hours spent in front of a screen per day. This was condensed into a categorical variable of screen time hours per day (*< 2 h/day*,* 2 to < 4 h/day*,* 4 to < 6 h/day*,* and ≥ 6 h/day)*. The reference group (< 2 h/day) was chosen as this is recommended in guidelines for adolescents’ daily screen time [16, 17].

### Physical activity

Information on physical activity during the preceding month was collected at age 11 based on the following questions: “How do you usually use your body during leisure time?” and “Do you participate in sport in your leisure time?”, which was followed by a question giving examples of sports such as soccer, basketball, volleyball, tennis and more, thus indicating organized team sports. Based on the answers to the two questions, we created a combined measure of physical activity level (*highest*,* moderate*,* light*,* and lowest*) (see Supplementary Fig. 1).

### Suicidality

At age 18 years and 3 months, adolescents were asked regarding lifetime history of suicidal ideation and suicide attempt with the response options: *yes*,* no*, and *do not know* (see Supplementary Table 2). Answers of *do not know* were categorized as *no*. In addition, information on all hospital-recorded suicide attempts prior to the date of completing the DNBC-18 was obtained from the National Patient Register and the Psychiatric Central Research Register, using a previously defined algorithm for probable suicide attempts, while omitting incidents recorded before the age of 10 as the majority of these have been identified as accidental poisonings among children [18]. Self-reported and register-based information on suicidality were combined into a hierarchical variable with three mutually exclusive categories of suicidal severity: *(1) no suicidality* (i.e. no suicidal ideation and no suicide attempt); *(2) suicidal ideation* (i.e. no suicide attempt); *(3) suicide attempt* (i.e. self-reported and/or hospital-recorded suicide attempt).

### Covariates

To account for potential confounding, information on the following self-reported covariates were derived from DNBC-11: alcohol experience, smoking experience, number of close friends, score from Stress in Children scale, depression symptoms, self-reported non-suicidal self-harm and sleep duration (Supplementary Table 3). Using register data, we additionally retrieved information on parental educational level, parental income level (categorized as income quartiles and based on yearly data to adjust for inflation), parental occupational status, family living situation, parental history of mental disorder, and adolescent’s history of mental disorder. Register-based data was derived for the year when the adolescent turned 11 years. Missing information on all covariates were very low (< 2.5%). In preliminary analyses their impact was examined as separate categories and found unsubstantial and consequently missing responses were placed in the most frequent category for each covariate.

### Applying weigths

To account for selection bias and improve generalizability of our results, we applied inverse probability weighting (IPW) using register-based data on the background population as reference. The background population was defined as all individuals who were born in Denmark from mid-1996 to mid-2003 and alive on their 18th birthday (*n* = 449 288). Each individual in the study population was assigned a weight representing themselves and others in the background population with same characteristics that may be over- or underrepresented [19]. The probability of participation in both DNBC-11 and DNBC-18 was estimated using register-based factors, of which some previously have been identified to predict selection into the DNBC cohort. Weights were formed based on following factors parental educational level, parental income level, parental occupational status, family living situation, parental history of mental disorder, adolescent’s history of mental disorder, maternal birth age, and parity and were used in all analyses.

### Statistical analysis

Associations between levels of screen time and physical activity and suicidality were examined using crude and adjusted relative risk ratios (aRRR) and their corresponding 95% confidence intervals (CI) using multinomial logistic regression models [20]. Statistical significance was determined by a p-value < 0.05 using two-sided tests. In addition to separate models of screen time and physical activity level, the exposures were examined jointly in a model with four categories: (1) low screen time and high physical activity, 2) high screen time and high physical activity, 3) low screen time and low physical activity, and 4) high screen time and low physical activity) where low screen time was defined as less than 4 h of screen time per day, and low physical activity as light or lowest level of physical activity. Adjusted models accounted for parental educational level, parental income level, parental occupational status, family living situation, parental history of mental disorder, adolescent’s alcohol experience, and adolescent’s smoking experience. Further analysis for screen time was adjusted for physical activity and vice versa. As preliminary findings had revealed an interaction between sex and physical activity (*p* = 0.016) and screen time (*p* < 0.001), analyses were conducted separately for males and females. The robustness of the estimates was evaluated in sensitivity analyses by excluding: (1) individuals who responded “*do not know*” to the questions regarding suicidality; (2) individuals who had reported previous deliberate self-harm at age 11; (3) individuals who reported suicidality in the year prior to answering DNBC-18 (to ensure that screen time and physical activity had been measured before the outcome occurred). Further sensitivity analyses consisted of (4) separately analyzing responses to the two questions regarding physical activity (“How do you usually use your body during leisure time?” and “Do you participate in sport in your leisure time?”) to determine whether they had an independent association with suicidality and (5) additional adjustment for indicators of child mental health (Stress in Children scale, self-reported non-suicidal self-harm, sleep duration, adolescent’s history of mental disorder) and social life (number of close friends) at age 11. These indicators were not included in the primary analyses as they were reported at the same time as the exposure variables, thus it was not possible to determine whether they represent mediators rather than confounders, i.e., consequently this model likely represent an over-adjustment.

Data management and statistical analyses were conducted in SAS version 9.4.

### Ethics

Anonymized data sets were used for the analyses, and the project was approved by the Danish Data Protection Agency (P-2020-305).

## Results

Sample weights were applied to align the DNBC population with the background population (Tables 1 and 2). At age 11, 25.2% of females and 15.2% of males reported to spend < 2 h per day on screen time, whereas 6.9% of the females and 12.6% of the males used ≥ 6 h. With regard to physical activity, 24.7% of the females and 29.7% of the males reported the highest physical activity level, while 13.4% of the females and 14.8% of the males reported the lowest physical activity level. In Supplementary Tables 4 and 5 characteristics are presented by level of screen time. The highest proportion of adolescents coming from homes characterized by lower parental income, lower parental education, divorced parents and parental psychiatric diagnosis were in the group of those with an average screen time of at least 6 h/daily.

**Table 1:** Characteristics among the DNBC population, the background population, and the weighted DNBC population for females

	DNBC population		Background population		Weighted DNBC population	
	N (%)		N (%)		N (%)	
Total	17 101		218 869		15 879	
Maternal birth age						
< 25 years	1177	(6.9)	32 857	(15.0)		(12.2)
25 to 29 years	6645	(38.9)	79 560	(36.4)		(36.5)
30 to 34 years	6547	(38.3)	74 259	(33.9)		(35.6)
≥ 35 years	2732	(16.0)	32 193	(14.7)		(15.6)
Parental educational level						
Elementary school	287	(1.7)	18 257	(8.3)		(5.0)
Vocational education	4201	(24.6)	75 844	(34.7)		(36.5)
Highschool education	919	(5.4)	14 798	(6.8)		(6.9)
Bachelor’s degree or higher	11 694	(68.4)	104 376	(47.7)		(51.6)
Missing			5594	(2.6)		
Parental income level						
1st quartile (lowest)	1787	(10.4)	56 498	(25.8)		(20.5)
2nd quartile	3798	(22.2)	54 223	(24.8)		(26.4)
3rd quartile	5292	(30.9)	54 073	(24.7)		(26.3)
4th quartile (highest)	6224	(36.4)	54 075	(24.7)		(26.7)
Parental occupational status						
Working/studying	15 399	(90.0)	164 396	(75.1)		(80.2)
Not working	1702	(10.0)	54 473	(24.9)		(19.8)
Family living situation						
Living with both parents	13 261	(77.5)	142 701	(65.2)		(68.2)
Not living with both parents	3840	(22.5)	76 168	(34.8)		(31.8)
Parental history of mental disorder						
No	15,627	(91.4)	186 737	(85.3)		(86.7)
Yes	1474	(8.6)	32 132	(14.7)		(13.3)
Adolescent’s history of mental disorder						
No	16 881	(98.7)	214 372	(97.9)		(98.1)
Yes	220	(1.3)	4497	(2.1)		(1.9)
Parity of mother						
1 child	8694	(50.8)	98 539	(45.0)		(45.8)
2 children	5905	(34.5)	79 151	(36.2)		(37.1)
≥ 3 children	2502	(14.6)	41 179	(18.8)		(17.1)

**Table 2:** Baseline characteristics among the DNBC population, the background population, and the weighted DNBC population for males

	DNBC population		Background population		Weighted DNBC population	
	N (%)		N (%)		N (%)	
Total	11 512		230 419		10 601	
Maternal birth age						
< 25 years	745	(6.5)	34 676	(15.0)		(12.1)
25 to 29 years	4409	(38.3)	83 284	(36.1)		(36.4)
30 to 34 years	4512	(39.2)	78 442	(34.0)		(36.0)
≥ 35 years	1846	(16.0)	34 017	(14.8)		(15.5)
Parental educational level						
Elementary school	154	(1.3)	19 270	(8.4)		(4.5)
Vocational education	2532	(22.0)	79 499	(34.5)		(36.5)
Highschool education	553	(4.8)	15 354	(6.7)		(6.7)
Bachelor’s degree or higher	8273	(71.9)	110 338	(47.9)		(52.3)
Missing			5958	(2.6)		
rental income level						
1st quartile (lowest)	1086	(9.4)	59 409	(25.8)		(20.1)
2nd quartile	2430	(21.1)	56 811	(24.7)		(26.4)
3rd quartile	3574	(31.0)	57 057	(24.8)		(26.6)
4th quartile (highest)	4422	(38.4)	57 142	(24.8)		(26.8)
Parental occupational status						
Working/studying	10 387	(90.2)	173 247	(75.2)		(80.9)
Not working	1125	(9.8)	57 172	(24.8)		(19.1)
Family living situation						
Living with both parents	9279	(80.6)	149 939	(65.1)		(68.6)
Not living with both parents	2233	(19.4)	80 480	(34.9)		(31.4)
Parental history of mental disorder						
No	15 627	(91.4)	196 638	(85.3)		(87.0)
Yes	1474	(8.6)	33 781	(14.7)		(13.0)
Adolescent’s history of mental disorder						
No	11,068	(96.1)	216 999	(94.2)		(94.4)
Yes	444	(3.9)	13 420	(5.8)		(5.6)
Parity of mother						
1 child	5935	(51.6)	103 527	(44.9)		(46.3)
2 children	3868	(33.6)	83 500	(36.2)		(37.0)
≥ 3 children	1709	(14.8)	43 392	(18.8)		(16.7)

### Screen time

**Fig. 2:**
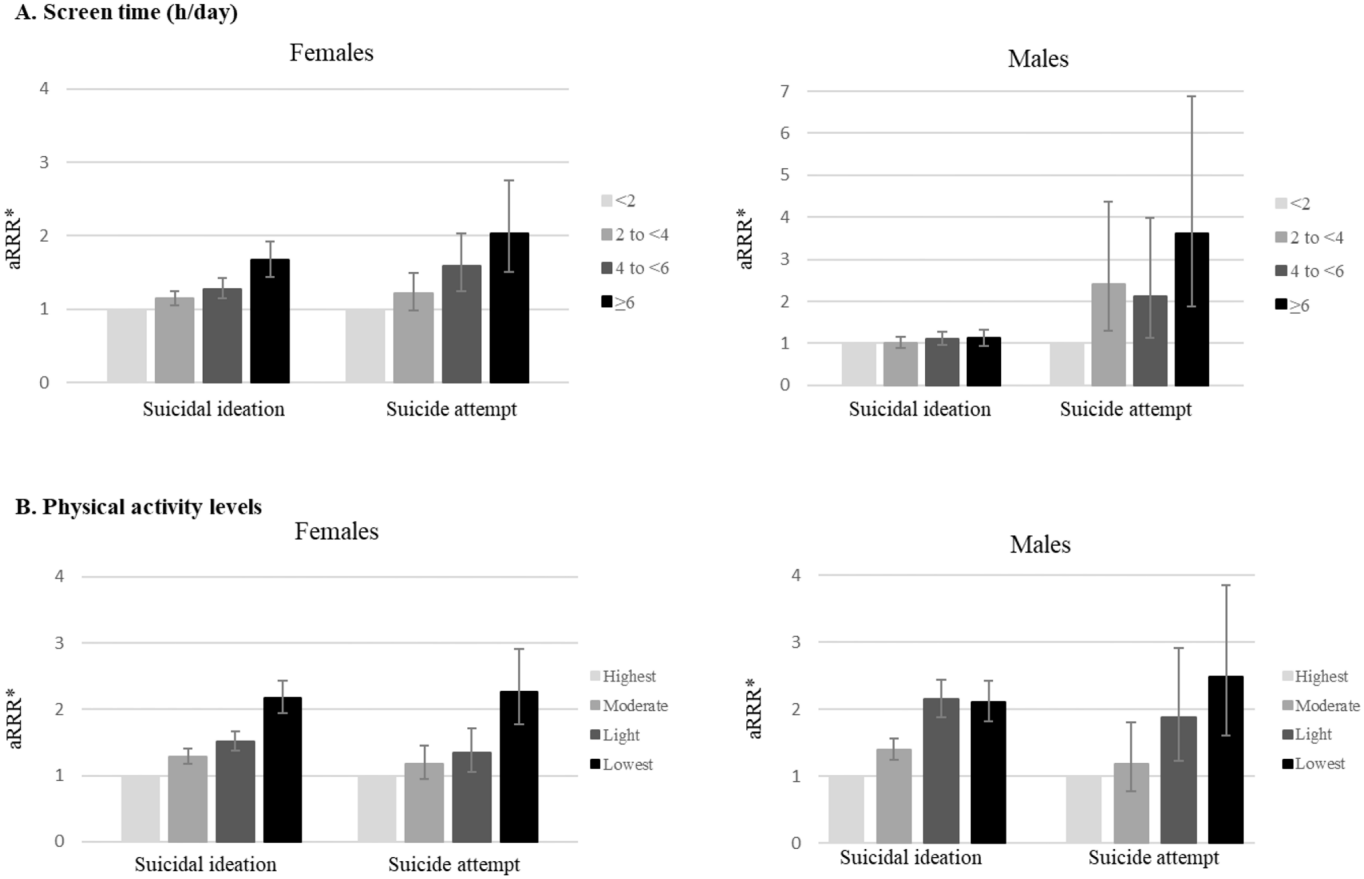
Associations between screen time and physical activity levels and suicidality in adolescent females and males^#†^. ^†^Estimates from main analysis (See Supplementary Table 6). ^#^Weighted estimates. ^*^Adjusted for parental educational level, parental income level, parental occupational status, family living situation, parental history of mental disorder, adolescent’s smoking experience, and adolescent’s alcohol experience (all at baseline), and the analysis for screen time is adjusted for physical activity levels, and the analysis for physical activity levels is adjusted for screen time

Compared to those with low screen time, i.e. <2 h per day, females with high screen time, i.e. ≥6 h, had an increased risk of suicidal ideation (aRRR: 1.67, 95% CI: 1.44–1.93), while no significant association was found for males (aRRR: 1.12, 95% CI: 0.94–1.32) (Fig. 2, Supplementary Table 6). Adolescents who reported high screen time had an increased risk of suicide attempt (females: aRRR: 2.04, 95% CI: 1.51–2.75; males: aRRR: 3.61, 95% CI: 1.89–6.89) when compared to those with low screen time. Overall, a dose response pattern indicated that higher levels of screen time were associated with higher aRRR estimates of suicide ideation and suicide attempt in females.

### Physical activity

Compared to individuals with the highest level of physical activity, those with the lowest level of physical activity had a higher risk of suicidal ideation (females: aRRR: 2.18, 95% CI: 1.94–2.44; males: aRRR: 2.11, 95% CI: 1.83–2.43) and suicide attempt (females: aRRR: 2.27, 95% CI: 1.77–2.91; males: aRRR: 2.49, 95% CI: 1.61–3.85). Overall, a dose response pattern was observed for both sexes, which indicated that lower levels of physical activity were linked to higher aRRR estimates for suicidal ideations and suicide attempts.

### Screen time and physical activity combined

Individuals with high screen time and low physical activity were found to have higher risk for both suicidal ideation (females; aRRR: 1.84, 95% CI: 1.66–2.05, males; aRRR: 1.94, 95% CI: 1.73–2.17) and suicide attempt (females; aRRR: 2.35, 95% CI: 1.89–2.91, males; aRRR: 2.70, 95% CI: 1.85–3.94) when compared to those with low screen time and high physical activity level (Table 3). Further, males who reported a low level of physical activity, independently of screen time level, were found to have markedly and significantly higher risk for suicide attempt compared to males categorized with a high level of physical activity (all p-values < 0.05). Thus, for example for males with high screen time and high physical activity level the relative risk were 1.73 (95% CI:1.14–2.62) compared with the reference group, while males with, respectively, low screen time and low physical activity as well as high screen time and low physical activity had relative risks on 2.73 (95% CI: 1.83–4.07) and 2.70 (95% CI: 1.85–3.95). The same pattern with markedly higher relative risks for suicide ideation in males with low physical activity levels, independently of screen time levels, was also found.

**Table 3:** Relative risk ratio of suicidality in relation to screen time and physical activity

	N	SI/SA n	Unadjusted^#^		Adjusted^#†^	
			Suicidal ideation RRR* (95% CI)	Suicide attempt RRR* (95% CI)	Suicidal ideation RRR* (95% CI)	Suicide attempt RRR* (95% CI)
Females						
Low screen time and high physical activity	9445	2812/268	1	1	1	1
High screen time and high physical activity	2198	792/85	1.37 (1.24 − 1.52)	1.59 (1.26 − 2.01)	1.32 (1.20 − 1.47)	1.36 (1.07 − 1.73)
Low screen time and low physical activity	3671	1490/140	1.62 (1.50 − 1.76)	1.76 (1.45 − 2.13)	1.53 (1.41 − 1.66)	1.43 (1.18 − 1.75)
High screen time and low physical activity	1787	794/108	1.99 (1.79 − 2.21)	3.21 (2.60 − 3.95)	1.84 (1.66 − 2.05)	2.35 (1.89 − 2.91)
Males						
Low screen time and high physical activity	5633	1247/51	1	1	1	1
High screen time and high physical activity	2179	542/32	1.18 (1.04 − 1.33)	1.86 (1.23 − 2.82)	1.19 (1.05 − 1.34)	1.73 (1.14 − 2.62)
Low screen time and low physical activity	1764	606/42	1.92 (1.70 − 2.16)	3.12 (2.10 − 4.63)	1.86 (1.64 − 2.10)	2.73 (1.83 − 4.07)
High screen time and low physical activity	1936	704/48	2.00 (1.79 − 2.24)	3.32 (2.29 − 4.81)	1.94 (1.73 − 2.17)	2.70 (1.85 − 3.94)

SI = suicidal ideation, SA = suicide attempt, RRR = Relative risk ratio

^**#**^Weighted estimates

^†^Adjusted for parental educational level, parental income level, parental occupational status, family living situation, parental history of mental

disorder, adolescent’s smoking experience, and adolescent’s alcohol experience

### Sensitivity analyses

When excluding those who answered ‘do not know’ regarding suicidality (supplementary Table 7) as well as those who self-reported deliberate self-harm at age 11 (supplementary Table 8) and those who reported suicidality prior to age 17 years and 3 months (supplementary Table 9), we obtained comparable estimates to those of the main analyses. When examining the items of physical activity separately, we found that both *team sport* and *leisure time physical activity* were associated with suicidal ideations and suicide attempt (supplementary Table 10). Finally, adjusting for indicators of child’s mental wellbeing and social life rendered comparable aRRR estimates, though slightly attenuated than those of the main analyses (supplementary Table 11).

## Discussion

Using a longitudinal study design with detailed self-reported data and national register data, we found that high levels of screen time were linked to elevated risks of suicidal ideation (for females) and suicide attempt. On the other hand, lower levels of physical were associated with elevated risks of suicidal ideation and suicide attempt. Seemingly, low levels of physical activity played a more important role for risks of suicidality, irrespective of level of screen time in particularly males.

In support of previously cross-sectional studies [4–8], we found that high screen time was associated with increased risks of suicidal ideation (females only) and suicide attempt. It should, however, be emphasized that screen time was assessed during 2010–2014; being the years where tablets were launched and use of smartphones/social media began to increase steeply [21], it is plausible that leisure screen time might be higher nowadays. The integration of tablets and computers in educational institutions has likely increased screen time exposure even further. Adverse effects of screen time include repeated exposure to idealized images, which might impact self-esteem and resilience and, thus, possibly also suicidality [22]. Another important factor is cyber bullying; those bullied online show greater levels of depression and loneliness, also linked to suicidality [23].

Our findings support existing evidence regarding low physical activity levels being associated with suicidality [5, 6, 8]. Although select cross-sectional studies [10–12] have reported an association between high levels of physical activity and suicidality for females, it was not found in this study. Type of physical activity might be more important than activity levels with respect to adolescents’ vulnerability towards suicidality.^9^ Team sports have been associated with lower odds of suicidality [24]; it is plausible that social and moral support provided by teammates and coaches might improve adolescents’ resilience and, thus, reduce suicidality [25]. Nevertheless, we found that both team sport and leisure time physical activity independently were significantly associated with suicidality in our sensitivity analyses.

Combinations of insufficient physical activity and high screen time have been linked to psychological difficulties among adolescents,^25^ which are associated with adolescent suicidality [26, 27, 28]. Our results furthermore indicated that any combination of screen time and high physical activity were associated with lower risk estimates for suicide ideation (for both sexes) and suicide attempt (for males) than any combination of screen time and low physical activity for males. This is supported by findings, which link physical activities to improved self-esteem, increased social interaction and fewer depressive symptoms and potentially boost resilience towards suicidality [29]. This may be important knowledge for future interventions aiming to prevent suicide as it indicates that it may be important to focus mostly on interventions aimed at adolescents’ physical activity levels rather than reducing screen time.

### Implications

As many as 74% of Danish adolescents aged 11–15 years do seemingly not comply with the recommended 60 min of moderate to vigorous physical activities on a daily basis [30], thus emphasizing the importance of this modifiable factor. Guidelines on screen time and daily physical activity for adolescents are generally disseminated by health authorities, and recently, the Danish Health Authority has released new guidelines regarding physical activity [31] and screen time [32], respectively. For the first time, the Danish Health Authority has suggested a time specific guideline for screen time (maximum 1–2 h per day for children and adolescents aged 5 to 17 years), whereas the guidelines for physical activity remain 60 min per day but with more specific guidelines on muscle strengthening activities. While these recommendations were not specifically designed and implemented from a suicide prevention perspective, the results of our paper suggest that following these guidelines may also have a preventive effect on suicidal thoughts and -behaviors. The implementation of guidelines for physical activity might improve through school-initiated activities. In 2014, a new Danish school policy requiring 45 min physical activity during school hours was implemented. This initiative increased levels of physical activity during school time, but no overall increase in physical activity through the whole day was observed [30]. This indicates that less physical activity occurs in leisure time which may be explained by more time used on screens. A recent meta-analysis on school-based interventions for physical activity and sedentary behavior found a beneficial effect on positive mental health, well-being and resilience, however authors also highlight the need for future studies of high-quality physical activity interventions in school context and a better understanding of mechanisms of how to implement these interventions before they can be used in practice [33]. Studies looking explicitly at interventions for reducing screen time in adolescents are generally lacking. Though, a reducing effect on screen time have been reported in an intervention targeting both adolescents and parents with components of an interactive seminar on self-monitoring of screen time, motivational messages and parental strategies for setting limits on screen viewing [34]. At follow-up this study however did not find an effect on mental health measures or physical activity levels between intervention- and control groups. Overall future studies examining effect (short and long-term) and feasibility of interventions aimed at increasing physical activity levels and decreasing screen time levels are still needed.

### Strengths and limitations

This study has several strengths. First, DNBC is a large nationwide cohort study, which was linked to data on the Danish background population, thus addressing any selection bias by applying weights and making estimates more generalizable. Linkage to individual-level register data on the adolescents and their parents in addition to self-reported data were applied as co-variates and lowered bias by potential confounders. Further, in contrast to most previous studies on this subject, this was a longitudinal designed study where the exposures were measured already at the age of 11 years and the outcome at age 18. It is reasonable to assume that the exposures preceded the outcome, as suicidal ideation and behaviours typically begins to show more prominently from the age of 13–14 years [35, 36].

Limitations should be mentioned. First, attrition occurred in the longitudinal design, and while we used weighting procedures there is no guarantee that we accounted for all selection bias. Second, self-reported data implied that information bias cannot be excluded. Third, a measure which captured the 60 min daily moderate to vigorous activities, as recommended by WHO, or actigraphy would have been preferred but was not available. Fourth, our measure of screen time was based on time spent on computer, TV and video, which were the commonly used screens when questions were prepared for the DNBC-11 follow-up carried out between 2010 and 2014. A screen measure including time spent on mobile phones and tablets would have fitted better for measuring screen time among adolescents as of present time. Further, it would have been a stronger screen time measure if it had also encapsulated information on screen content and not merely screen time. Fifth, we applied a crude measure for self-reported suicidal ideation and -attempt, i.e. not a validated scale. Sixth, data on exposures and confounders was collected at the same time, thus, precluding the possibility of determining whether one type of exposure had predated the other type of exposure or confounder.

In sum, our results show that comparatively higher levels of screen time and lower levels of physical activity reported by 11-year-old adolescents was associated with increased risks for lifetime suicidality reported at age 18. Being modifiable factors, emphasize the importance of taking efforts of decreasing screen time and increasing physical activity into consideration when designing suicide preventive efforts for adolescents.

## Electronic supplementary material

Below is the link to the electronic supplementary material.

Supplementary Material 1

## Data Availability

Data availability: The data that support the findings of this study are available from Statistics Denmark and from Statens Serum Institut. Data access requires the completion of a detailed application form from the Danish Data Protection Agency, the Danish National Board of Health and Statistics Denmark and from Statens Serum Institut. For more information on accessing the data, see https://www.dst.dk/en and https://www.dnbc.dk.
