# Supplementary material for: Associations of screen time and physical activity with suicidality in adolescents: a national cohort study
Source: Soc Psychiatry Psychiatr Epidemiol. 2025 Feb 17;60(7):1639–48. doi: 10.1007/s00127-025-02827-1 (PMC12238212; doi:10.1007/s00127-025-02827-1)
Supplement: Supplementary file 1 — Supplementary Material 1 [file 127_2025_2827_MOESM1_ESM.docx]

**eTable 1:** Questions related to exposure in DNBC-11.

| **Exposure** | **Question** | **Answer options** |
| --- | --- | --- |
| Screen time (computer on weekdays) | How much of your leisure time do you spend in front of the computer? Count all the time you sit in front of a computer. | - 0-½ hour  - ½ -1 hour  - 1-2 hours  - 2-3 hours  - 3-4 hours  - 4-5 hours  - >5 hours |
| Screen time (computer on weekend days) | How much of your leisure time do you spend in front of the computer? Count all the time you sit in front of a computer. | - 0-½ hour  - ½ -1 hour  - 1-2 hours  - 2-3 hours  - 3-4 hours  - 4-5 hours  - 5-6 hours  - 6-7 hours  - 7-8 hours  - >8 hours |
| Screen time (TV/DVD/Video on weekdays) | How much of your leisure time do you spend watching tv? (include DVD/video or watching films on your computer). | - 0-½ hour  - ½ -1 hour  - 1-2 hours  - 2-3 hours  - 3-4 hours  - 4-5 hours  - >5 hours |
| Screen time (TV/DVD/Video on weekend days) | How much of your leisure time do you spend watching tv? (include DVD/video or watching films on your computer). | - 0-½ hour  - ½ -1 hour  - 1-2 hours  - 2-3 hours  - 3-4 hours  - 4-5 hours  - 5-6 hours  - 6-7 hours  - 7-8 hours  - >8 hours |
| Physical activity in leisure time | How do you usually use your body during your leisure time? Think about the last month when choosing your answer | - I am very active: I do sports, I run around or play ball most of the time.  - I am pretty active: I run around a lot, I do sports or play wildly most of the time.  - I am a little active: I play with my friends and walk around most of the time.  - I am not active: I sit and talk, watch TV or read, play games or computer games most of my leisure time. |
| Engagement in sport in leisure time | Do you participate in sport in your leisure time? | - Yes  - No |

**eTable 2**: Measurements of suicide ideation and suicide attempt from DNBC-18 and register-based

hospital contacts for probable suicide attempt.

| **Outcome** | **Question** | **Answer options** |
| --- | --- | --- |
| Suicidal ideation  (self-reported from DNBC-18) | Have you ever thought about taking your own life (even though you would not do it)? | 0. No  1. Yes  2. Do not know |
| Suicide attempt  (self-reported from DNBC-18) | Have you ever tried to kill yourself? | 0. No  1. Yes  2. Do not know |
| Suicide attempt  (register-based hospital contacts for probable suicide attempts)^1,2^ | - All hospital contacts with a main or supplementary ICD-10 diagnosis of X60-X84 or where reason of contact code for “suicide attempt”.  - Main diagnosis accidental intoxication with weak analgesic drugs, antiepileptics, sleeping pills, antiparkinsonian drugs, psychotropics, and carbon monoxide (ICD-10: T39, T40 except T40.1, T42, T43, T58).  - Main diagnosis of psychiatric disorder diagnosis (ICD-10: F00-F99) in combination with a sub-diagnosis of intoxication with drugs and biological substances (ICD-10: T36-T50, T52-T60).  - Main diagnosis of psychiatric disorder diagnosis (ICD-10: F00-F99) in combination with a sub-diagnosis of injuries to the lower forearm (ICD-10: S51, S55, S59, S61, S65, S69). | |

**^1^** Mors O, Perto GP, Mortensen PB. The Danish psychiatric central research register. *Scand J Public Health*. 2011;39(7):54-57.

**^2^** Lynge E, Lynge Sandegaard J, Rebolj M. The Danish National Patient Register. *Scand J Public Health*. 2011;39(7):30-33

**eTable 3:** Overview of included covariates.

| **Variable** | **Categories** | **Description** | **Data source** |
| --- | --- | --- | --- |
| **Maternal birth age**^†^ | - < 25 years - 25-29 years - 30-34 years - ≥35 years | Mothers age at birth of child, who participated in DNBC | The Medical Birth Register^1^ |
| **Parity**^†^ | - 1 child - 2 children - ≥3 children | Number of mother’s past liveborn children at the time of giving birth to the child participating in DNBC | The Medical Birth Register^1^ |
| **Parental educational level** | - Elementary school (9 to 10 years in school) - Vocational training - High school education - Bachelor's degree or higher | Highest obtained educational degree was of any parent, which had been attained the year of the adolescent’s 11th birthday. | The Education Register^2^ |
| **Parental income level** | - 1st income quartile (lowest) - 2nd income quartile - 3rd income quartile - 4th income quartile (highest) | The highest income level of any parent(s) in the year of the adolescent’s 11th birthday. | The Income Statistics Register^3^ |
| **Parental occupational status** | - Working (including studying and retired) - Not working (including unemployed, sickness leave, leave of absence, early retirement, and other transfer income) | Based on socioeconomic classification, a binary outcome was created. Measured the year of adolescent’s 11^th^ birthday. | The Employment Classification Module^4^ |
| **Family living situation** | - Living with both parents - Not living with both parents | Information on family type was obtained and categorised as a binary covariate based on range of family types. | The Civil Registration System^5^ |
| **Parental history of mental disorder** | - No - Yes | Mental disorders were recorded according to the 10^th^ revision of the International Classification of diseases (ICD-10) as any diagnose from the ‘F-chapter’ (F00-F99). | The Psychiatric Central Research Register^6^ |
| **Adolescent’s history of mental disorder** | - No - Yes | Mental disorders were recorded according to the 10^th^ revision of the International Classification of diseases (ICD-10) as any diagnose from the ‘F-chapter’ (F00-F99). | The Psychiatric Central Research Register^6^ |
| **Smoking experience** | - Never tried to smoke an entire cigarette - Ever tried to smoke an entire cigarette | Based on a question whether they had ever tried to smoke an entire cigarette, which could be answered with “yes” and “no”. | DNBC-11 |
| **Alcohol experience** | - Never tried to drink more than one unit of alcohol - Ever tried to drink more than one unit of alcohol | Based on a question whether they had ever tried to drink more than one alcopop, one beer, a glass of wine or a glass of spirits, which could be answered with “yes” and “no”. | DNBC-11 |
| **Stress in Children’s scale (SiC)*** | - No stress (score: <2) - Medium stress (score: 2 to <2.5) - High stress (score: ≥2.5) | SiC is a validated questionnaire with 21 statements (including information on loneliness, bullying, adult support, and more) and four possible response options for each statement (very often, often, sometimes, never). The score was calculated as the sum of scores for individual answers and divided by the number of statements, thus, providing an average score, as advised by the author of the questionnaire.^7,8^ | DNBC-11 |
| **Deliberate self-harm (DSH)*** | - Never done or thought of DSH - Ever done or thought of DSH | Based on three questions related to DSH with the response categories “yes” and “no”. If the adolescent had answered yes to just one of the questions they were considered as having “ever done or thought of DSH” as advised by the Development and Well-Being Assessment. ^9^ | DNBC-11 |
| **Number of close friends*** | - 0 friends - 1 friend - 2 friends - ≥3 friends | Based on two questions related to the current number of close male friends/female friends, which each had four response categories, the total number of friends was derived by adding male and female friends. | DNBC-11 |
| **Sleep*** | - <9 hours each night - ≥9 hours each night | Based on questions regarding what time they went to sleep and at what time they would wake up, the average number of sleeping hours was calculated as the sum of the number of hours of sleep before midnight and after midnight. A 9-hours cut-off was used based on the general sleep recommendation for adolescents of 11 years of age.^10^ | DNBC-11 |
| **Depression** | - Yes (with depressive symptoms) - No (without depressive symptoms) | Based on replies to three questions in *The development and Well-being Assessment scale. 1)* “During the last 4 weeks have there been times when you have been very sad, miserable, unhappy or tearful?”, 2) “During the last 4 weeks has there been a period when you have been really miserable nearly every day?” and 3) “During the time when you have been miserable, have you been really miserable for most of the day?” If the child reported ‘yes’ to all three questions it was categorized as ‘with depressive symptoms’. | DNBC-11 |

*Only included in sensitivity analyses
^†^ Only included in inverse probability weighting procedure

1. Bliddal M, Broe A, Pottegård A, Olsen J, Langhoff-Roos J. The Danish Medical Birth Register. *Eur J Epidemiol*. 2018;33(1):27-36. doi:10.1007/S10654-018-0356-1/TABLES/5

2. Jensen VM, Rasmussen AW. Danish education registers. *Scand J Public Health*. 2011;39(7):91-94. doi:10.1177/1403494810394715

3. Baadsgaard M, Quitzau J. Danish registers on personal income and transfer payments. *Scand J Public Health*. 2011;39(7):103-105. doi:10.1177/1403494811405098

4. Petersson F, Baadsgaard M, Thygesen LC. Danish registers on personal labour market affiliation. *Scand J Public Health*. 2011;39(7):95-98. doi:10.1177/1403494811408483

5. Pedersen CB. The Danish civil registration system. *Scand J Public Health*. 2011;39(7):22-25. doi:10.1177/1403494810387965Lynge E, Lynge Sandegaard J, Rebolj M. The Danish National Patient Register. *Scand J Public Health*. 2011;39(7):30-33. doi:10.1177/1403494811401482

6. Mors O, Perto GP, Mortensen PB. The Danish psychiatric central research register. *Scand J Public Health*. 2011;39(7):54-57. doi:10.1177/1403494810395825

**7**. Osika W, Friberg P, Wahrborg P. A New Short Self-Rating Questionnaire to Assess Stress in Children. *Int J Behav Med Copyr C*. 2007;14(2):108-117.

8. Stallknecht SE, Strandberg-Larsen K, Hestbæk L, Andersen AMN. Spinal pain and co-occurrence with stress and general well-being among young adolescents: a study within the Danish National Birth Cohort. *Eur J Pediatr*. 2017;176(6):807-814. doi:10.1007/S00431-017-2915-Y

9. DAWBA. View PDFs with indicative DAWBA content. Published 2017. Accessed March 16, 2023. https://dawba.info/py/dawbainfo/b1.py

10. Sleep in Middle and High School Students | Healthy Schools | CDC. Accessed April 3, 2023. <https://www.cdc.gov/healthyschools/features/students-sleep.htm>

11. Goodman, R., Ford, T., Richards, H., Gatward, R., & Meltzer, H. (2000). The Development and Well-Being Assessment: Description and Initial Validation of

an Integrated Assessment of Child and Adolescent Psychopathology. Journal of Child Psychology and Psychiatry, 41(5), 645–655.

| **eTable 4: Characteristics of the weighted DNBC study population according to screen-time sedentary behaviour for males** | | | | | | | | | | | |
| --- | --- | --- | --- | --- | --- | --- | --- | --- | --- | --- | --- |
|  | |  | | **Average time spent on screen time (h/day)** | | | | | | | |
| **Characteristics** | | **N** | | **<2** | | **2 to <4** | | **4 to <6** | | **≥6** | |
| **Total** | | 11512 | | 15.8 | | 45.4 | | 26.2 | | 12.6 | |
| **PA levels** | |  | |  | |  | |  | |  | |
| Highest PA level | | 3671 | | 46.3 | | 33.4 | | 20.9 | | 13.7 | |
| Moderate PA level | | 4141 | | 32.7 | | 38.1 | | 34.1 | | 23.2 | |
| Light PA level | | 2354 | | 12.7 | | 17.4 | | 27.7 | | 31.9 | |
| Lowest PA level | | 1346 | | 8.3 | | 11.1 | | 17.3 | | 31.2 | |
| **Parental educational level** | |  | |  | |  | |  | |  | |
| Bachelor's degree or higher | | 8273 | | 58.6 | | 54.3 | | 50.4 | | 41.1 | |
| High school education | | 553 | | 6.7 | | 6.8 | | 6.4 | | 7.2 | |
| Vocational training | | 2532 | | 30.5 | | 34.9 | | 38.8 | | 44.5 | |
| Elementary school | | 154 | | 4.2 | | 4.0 | | 4.4 | | 7.2 | |
| **Household income** | |  | |  | |  | |  | |  | |
| 4th income quartile (highest) | | 4422 | | 30.7 | | 28.8 | | 25.0 | | 18.5 | |
| 3rd income quartile | | 3574 | | 25.9 | | 27.0 | | 27.2 | | 25.0 | |
| 2nd income quartile | | 2430 | | 24.5 | | 26.5 | | 25.6 | | 30.5 | |
| 1st income quartile (lowest) | | 1086 | | 18.9 | | 17.7 | | 22.3 | | 26.0 | |
| **Parental occupational status** | |  | |  | |  | |  | |  | |
| Working | | 10387 | | 82.5 | | 82.4 | | 80.5 | | 74.0 | |
| Not working | | 1125 | | 17.5 | | 17.6 | | 19.5 | | 26.0 | |
| **Family type** | |  | |  | |  | |  | |  | |
| Living with both parents | | 9279 | | 70.9 | | 69.5 | | 68.2 | | 63.7 | |
| Not living with (both) parents | | 2233 | | 29.1 | | 30.5 | | 31.8 | | 36.3 | |
| **Parental psychiatric diagnosis** | |  | |  | |  | |  | |  | |
| Not having any psychiatric diagnosis | | 10563 | | 86.7 | | 88.9 | | 86.0 | | 82.5 | |
| Having any psychiatric diagnosis | | 949 | | 13.3 | | 11.1 | | 14.0 | | 17.5 | |
| **Child's psychiatric diagnosis** | |  | |  | |  | |  | |  | |
| Not having any psychiatric diagnosis | | 11068 | | 94.3 | | 96.2 | | 93.4 | | 90.4 | |
| Having any psychiatric diagnosis | | 444 | | 5.7 | | 3.8 | | 6.6 | | 9.6 | |
| **SiC** | |  | |  | |  | |  | |  | |
| No stress | | 7946 | | 74.5 | | 68.6 | | 62.3 | | 51.2 | |
| Medium stress | | 3209 | | 23.5 | | 27.9 | | 33.3 | | 41.4 | |
| High stress | | 357 | | 2.0 | | 3.5 | | 4.4 | | 7.4 | |
| **Number of close friends** | |  | |  | |  | |  | |  | |
| 3 friends or more | | 9723 | | 85.7 | | 85.2 | | 82.9 | | 80.0 | |
| 2 friends | | 1110 | | 9.0 | | 9.2 | | 10.6 | | 11.9 | |
| 1friend | | 529 | | 3.8 | | 4.4 | | 5.4 | | 5.8 | |
| 0 friends | | 150 | | 1.5 | | 1.3 | | 1.2 | | 2.3 | |
| **Sleep** | |  | |  | |  | |  | |  | |
| Sleeping ≥9 hours each night | | 10376 | | 95.3 | | 90.7 | | 85.9 | | 77.7 | |
| Sleeping <9 hours each night | | 1136 | | 4.7 | | 9.3 | | 14.1 | | 22.3 | |
| **DSH** | |  | |  | |  | |  | |  | |
| Never done or thought of DSH | | 10459 | | 91.8 | | 91.5 | | 89.1 | | 86.1 | |
| Ever done or thought of DSH | | 888 | | 5.9 | | 7.1 | | 9.2 | | 12.2 | |
| Missing | | 165 | | 2.3 | | 1.4 | | 1.7 | | 1.7 | |
| **Depression symptoms** | |  | |  | |  | |  | |  | |
| No | | 11171 | | 96.6 | | 96.4 | | 97.0 | | 96.5 | |
| Yes | | 140 | | 1.4 | | 1.2 | | 1.3 | | 1.5 | |
| Missing | | 201 | | 2.0 | | 2.4 | | 1.7 | | 2.0 | |
| **Child's smoking experience** | |  | |  | |  | |  | |  | |
| Never tried to smoke an entire cigarette | | 11442 | | 99.4 | | 99.5 | | 99.2 | | 97.9 | |
| Ever tried to smoke an entire cigarette | | 70 | | 0.6 | | 0.5 | | 0.8 | | 2.1 | |
| **Child's alcohol experience** | |  | |  | |  | |  | |  | |
| Never tried to drink more than one unit of alcohol | | 11172 | | 98.1 | | 97.0 | | 95.9 | | 91.1 | |
| Ever tried to drink more than one unit of alcohol | | 340 | | 1.9 | | 3.0 | | 4.1 | | 8.9 | |
| **eTable 5: Characteristics of the weighted DNBC study population according to screen-time sedentary behaviour for females** | | | | | | | | | | |  |
|  |  | | **Average time spent on screen time (h/day)** | | | | | | | |  |
| **Characteristics** | **N** | | **<2** | | **2 to <4** | | **4 to <6** | | **≥6** | |  |
| **Total** | 17101 | | 25.2 | | 49.2 | | 18.7 | | 6.9 | |  |
| **PA levels** |  | |  | |  | |  | |  | |  |
| Highest PA level | 4427 | | 34.5 | | 24.1 | | 15.9 | | 16.7 | |  |
| Moderate PA level | 7216 | | 40.1 | | 42.3 | | 38.5 | | 28.7 | |  |
| Light PA level | 3592 | | 16.8 | | 21.0 | | 28.1 | | 28.6 | |  |
| Lowest PA level | 1866 | | 8.7 | | 12.6 | | 17.5 | | 26.0 | |  |
| **Parental educational level** |  | |  | |  | |  | |  | |  |
| Bachelor's degree or higher | 11694 | | 59.5 | | 51.6 | | 45.8 | | 37.9 | |  |
| High school education | 919 | | 5.8 | | 6.7 | | 8.2 | | 8.9 | |  |
| Vocational training | 4201 | | 30.7 | | 37.2 | | 40.0 | | 43.5 | |  |
| Elementary school | 287 | | 3.9 | | 4.6 | | 6.0 | | 9.6 | |  |
| **Household income** |  | |  | |  | |  | |  | |  |
| 4th income quartile (highest) | 6224 | | 30.5 | | 27.0 | | 23.2 | | 20.3 | |  |
| 3rd income quartile | 5292 | | 27.0 | | 26.9 | | 25.2 | | 23.2 | |  |
| 2nd income quartile | 3798 | | 22.3 | | 26.5 | | 30.5 | | 29.2 | |  |
| 1st income quartile (lowest) | 1787 | | 20.2 | | 19.6 | | 21.0 | | 27.3 | |  |
| **Parental occupational status** |  | |  | |  | |  | |  | |  |
| Working | 15399 | | 81.1 | | 81.6 | | 77.6 | | 74.1 | |  |
| Not working | 1702 | | 18.9 | | 18.4 | | 22.4 | | 25.9 | |  |
| **Family type** |  | |  | |  | |  | |  | |  |
| Living with both parents | 13261 | | 70.3 | | 69.3 | | 65.7 | | 58.8 | |  |
| Not living with (both) parents | 3840 | | 29.7 | | 30.7 | | 34.3 | | 41.2 | |  |
| **Parental psychiatric diagnosis** |  | |  | |  | |  | |  | |  |
| Not having any psychiatric diagnosis | 15627 | | 88.5 | | 86.5 | | 85.9 | | 83.8 | |  |
| Having any psychiatric diagnosis | 1474 | | 11.5 | | 13.5 | | 14.1 | | 16.2 | |  |
| **Child's psychiatric diagnosis** |  | |  | |  | |  | |  | |  |
| Not having any psychiatric diagnosis | 16881 | | 98.2 | | 98.2 | | 98.2 | | 96.9 | |  |
| Having any psychiatric diagnosis | 220 | | 1.8 | | 1.8 | | 1.8 | | 3.1 | |  |
| **SiC** |  | |  | |  | |  | |  | |  |
| No stress | 11847 | | 73.9 | | 67.8 | | 60.1 | | 48.3 | |  |
| Medium stress | 4571 | | 23.0 | | 28.1 | | 33.6 | | 38.3 | |  |
| High stress | 683 | | 3.1 | | 4.1 | | 6.3 | | 13.4 | |  |
| **Number of close friends** |  | |  | |  | |  | |  | |  |
| 3 friends or more | 15258 | | 88.6 | | 89.4 | | 89.2 | | 87.1 | |  |
| 2 friends | 1233 | | 7.7 | | 7.1 | | 7.1 | | 8.1 | |  |
| 1friend | 501 | | 3.1 | | 2.8 | | 3.1 | | 3.9 | |  |
| 0 friends | 109 | | 0.6 | | 0.7 | | 0.6 | | 0.9 | |  |
| **Sleep** |  | |  | |  | |  | |  | |  |
| Sleeping ≥9 hours each night | 15125 | | 92.6 | | 88.2 | | 82.1 | | 71.7 | |  |
| Sleeping <9 hours each night | 1976 | | 7.4 | | 11.8 | | 17.9 | | 28.3 | |  |
| **DSH** |  | |  | |  | |  | |  | |  |
| Never done or thought of DSH | 15590 | | 92.1 | | 91.5 | | 88.9 | | 79.8 | |  |
| Ever done or thought of DSH | 1300 | | 6.4 | | 7.3 | | 9.7 | | 18.6 | |  |
| Missing | 211 | | 1.5 | | 1.2 | | 1.3 | | 1.6 | |  |
| **Depression symptoms** |  | |  | |  | |  | |  | |  |
| No | 16343 | | 95.9 | | 96.1 | | 93.5 | | 90.2 | |  |
| Yes | 486 | | 2.3 | | 2.5 | | 4.4 | | 8.1 | |  |
| Missing | 272 | | 1.8 | | 1.4 | | 2.1 | | 1.7 | |  |
| **Child's smoking experience** |  | |  | |  | |  | |  | |  |
| Never tried to smoke an entire cigarette | 17031 | | 99.6 | | 99.6 | | 99.3 | | 97.4 | |  |
| Ever tried to smoke an entire cigarette | 70 | | 0.4 | | 0.4 | | 0.7 | | 2.6 | |  |
| **Child's alcohol experience** |  | |  | |  | |  | |  | |  |
| Never tried to drink more than one unit of alcohol | 16800 | | 98.7 | | 98.3 | | 97.0 | | 91.5 | |  |
| Ever tried to drink more than one unit of alcohol | 301 | | 1.3 | | 1.7 | | 3.0 | | 8.5 | |  |

**eTable 6:** Relative risk ratio of suicidal levels according to screen time and physical activity levels among the weighted

population of females and males, respectively.

|  |  |  | Unadjusted^#^ | | Adjusted^#†^ | |
| --- | --- | --- | --- | --- | --- | --- |
|  | N | **SI/SA**  n | **Suicidal ideation**  RRR (95% CI) | **Suicide attempt**  RRR (95% CI) | **Suicidal ideation**  RRR (95% CI) | **Suicide attempt**  RRR (95% CI) |
| **Screen time (hours per day)**  Females |  |  |  |  |  |  |
| <2 | 4614 | 1403/127 | 1 | 1 | 1 | 1 |
| - 2 to <4 | 8502 | 2899/281 | 1.21 (1.12-1.32) | 1.32 (1.07-1.62) | 1.15 (1.05-1.25) | 1.22 (0.99-1.50) |
| - 4 to <6 | 2999 | 1146/131 | 1.45 (1.32-1.61) | 1.98 (1.57-2.50) | 1.28 (1.15-1.42) | 1.59 (1.25-2.03) |
| - ≥6 | 986 | 440/62 | 2.05 (1.78-2.37) | 3.29 (2.47-4.37) | 1.67 (1.44-1.93) | 2.04 (1.51-2.75) |
| Males |  |  |  |  |  |  |
| - <2 | 1976 | 465/14 | 1 | 1 | 1 | 1 |
| - 2 to <4 | 5421 | 1388/79 | 1.08 (0.95-1.23) | 2.60 (1.43-4.72) | 1.01 (0.89-1.15) | 2.40 (1.31-4.36) |
| - 4 to <6 | 2897 | 843/46 | 1.32 (1.15-1.51) | 2.69 (1.44-5.03) | 1.10 (0.96-1.27) | 2.11 (1.12-3.97) |
| - ≥6 | 1218 | 403/34 | 1.49 (1.27-1.75) | 6.01 (3.22-11.22) | 1.12 (0.94-1.32) | 3.61 (1.89-6.89) |
| **Physical activity level**  Females |  |  |  |  |  |  |
| - Highest | 4427 | 1224/119 | 1 | 1 | 1 | 1 |
| - Moderate | 7216 | 2380/234 | 1.32 (1.21-1.45) | 1.23 (0.99-1.53) | 1.29 (1.18-1.41) | 1.17 (0.94-1.46) |
| - Light | 3592 | 1404/138 | 1.65 (1.50-1.83) | 1.68 (1.33-2.13) | 1.51 (1.37-1.67) | 1.35 (1.06-1.72) |
| - Lowest | 1866 | 880/110 | 2.53 (2.26-2.84) | 3.51 (2.76-4.45) | 2.18 (1.94-2.44) | 2.27 (1.77-2.91) |
| Males |  |  |  |  |  |  |
| - Highest | 3671 | 692/32 | 1 | 1 | 1 | 1 |
| - Moderate | 4141 | 1097/51 | 1.43 (1.28-1.61) | 1.30 (0.86-1.97) | 1.40 (1.25-1.57) | 1.19 (0.78-1.81) |
| - Light | 2354 | 834/48 | 2.29 (2.02-2.59) | 2.48 (1.64-3.76) | 2.15 (1.89-2.44) | 1.89 (1.23-2.91) |
| - Lowest | 1346 | 476/42 | 2.27 (1.98-2.60) | 3.71 (2.46-5.62) | 2.11 (1.83-2.43) | 2.49 (1.61-3.85) |

SI=Suicidal ideation, SA=Suicide attempt, RRR=Relative risk ratio.

**^#^**Weighted estimates

^†^Adjusted for parental educational level, parental income level, parental occupational status, family living situation, parental history of mental

disorder, adolescent’s smoking experience, adolescent’s alcohol experience, and the analysis for screen time is adjusted for physical activity

levels and the analysis for physical activity levels is adjusted for screen time.

**eTable 7:** Relative risk ratio of suicidal levels according to a sub-population (n(females)=16,293, n(males)=11,066) where

participants answering “do not know” to questions related to outcome in DNBC-18 are removed from the analyses.

|  |  |  | Unadjusted^#^ | | Adjusted^#†^ | |
| --- | --- | --- | --- | --- | --- | --- |
|  | **N** | **SI/SA**  **n** | **Suicidal ideation**  **RRR (95% CI)** | **Suicide attempt**  RRR (95% CI) | **Suicidal ideation**  RRR (95% CI) | **Suicide attempt**  RRR (95% CI) |
| **Screen time (hours per day)**  Females |  |  |  |  |  |  |
| - <2 | 4394 | 1403/127 | 1 | 1 | 1 | 1 |
| - 2 to <4 | 8113 | 2899/281 | 1.21 (1.12-1.32) | 1.32 (1.07-1.62) | 1.15 (1.05-1.25) | 1.22 (0.99-1.51) |
| - 4 to <6 | 2848 | 1146/131 | 1.49 (1.34-1.65) | 2.02 (1.60-2.56) | 1.31 (1.18-1.46) | 1.64 (1.28-2.08) |
| - ≥6 | 938 | 440/62 | 2.18 (1.88-2.52) | 3.48 (2.61-4.65) | 1.77 (1.52-2.06) | 2.18 (1.61-2.96) |
| Males |  |  |  |  |  |  |
| - <2 | 1895 | 465/14 | 1 | 1 | 1 | 1 |
| - 2 to <4 | 5217 | 1388/79 | 1.08 (0.95-1.23) | 2.60 (1.43-4.72) | 1.00 (0.88-1.14) | 2.38 (1.31-4.34) |
| - 4 to <6 | 2787 | 843/46 | 1.32 (1.15-1.51) | 2.70 (1.44-5.03) | 1.09 (0.95-1.26) | 2.08 (1.11-3.93) |
| - ≥6 | 1167 | 403/34 | 1.51 (1.29-1.78) | 6.08 (3.26-11.37) | 1.11 (0.93-1.31) | 3.57 (1.87-6.82) |
| **Physical activity level**  Females |  |  |  |  |  |  |
| - Highest | 4228 | 1224/119 | 1 | 1 | 1 | 1 |
| - Moderate | 6872 | 2380/234 | 1.34 (1.22-1.46) | 1.24 (1.00-1.54) | 1.30 (1.19-1.42) | 1.18 (0.95-1.47) |
| - Light | 3409 | 1404/138 | 1.71 (1.54-1.89) | 1.73 (1.37-2.19) | 1.55 (1.40-1.72) | 1.39 (1.09-1.77) |
| - Lowest | 1784 | 880/110 | 2.60 (2.32-2.92) | 3.60 (2.84-4.57) | 2.21 (1.97-2.49) | 2.29 (1.79-2.95) |
| Males |  |  |  |  |  |  |
| - Highest | 3567 | 692/32 | 1 | 1 | 1 | 1 |
| - Moderate | 3966 | 1097/51 | 1.46 (1.31-1.64) | 1.33 (0.88-2.02) | 1.43 (1.28-1.61) | 1.21 (0.80-1.84) |
| - Light | 2266 | 834/48 | 2.35 (2.08-2.66) | 2.55 (1.68-3.87) | 2.21 (1.94-2.51) | 1.93 (1.26-2.97) |
| - Lowest | 1267 | 476/42 | 2.41 (2.10-2.76) | 3.94 (2.60-5.96) | 2.24 (1.93-2.58) | 2.63 (1.70-4.08) |

SI=Suicidal ideation, SA=Suicide attempt, RRR=relative risk ratio.

**^#^**Weighted estimates

^†^Adjusted for parental educational level, parental income level, parental occupational status, family living situation, parental history of mental

disorder, adolescent’s smoking experience, adolescent’s alcohol experience, and the analysis for screen time is adjusted for physical activity

levels and the analysis for physical activity levels is adjusted for screen time.

**eTable 8:** Relative risk ratio of suicidal levels according to a sub-population (n(females)=15,801, n(males)=10,624) excluding

those reporting DSH at age 11 for females and males, respectively.

|  |  |  | Unadjusted^#^ | | Adjusted^#†^ | |
| --- | --- | --- | --- | --- | --- | --- |
|  | **N** | **SI/SA**  **n** | **Suicidal ideation**  **RRR (95% CI)** | **Suicide attempt**  RRR (95% CI) | **Suicidal ideation**  RRR (95% CI) | **Suicide attempt**  RRR (95% CI) |
| **Screen time (hours per day)**  Females |  |  |  |  |  |  |
| - <2 | 4346 | 1285/91 | 1 | 1 | 1 | 1 |
| - 2 to <4 | 7926 | 2581/227 | 1.17 (1.07-1.27) | 1.43 (1.13-1.81) | 1.10 (1.01-1.20) | 1.28 (1.01-1.63) |
| - 4 to <6 | 2708 | 980/97 | 1.37 (1.23-1.52) | 2.07 (1.58-2.72) | 1.21 (1.08-1.35) | 1.66 (1.26-2.20) |
| - ≥6 | 821 | 345/38 | 1.85 (1.59-2.16) | 2.68 (1.87-3.83) | 1.54 (1.32-1.80) | 1.73 (1.19-2.53) |
| Males |  |  |  |  |  |  |
| - <2 | 1867 | 423/11 | 1 | 1 | 1 | 1 |
| - 2 to <4 | 5050 | 1218/58 | 1.09 (0.95-1.25) | 2.40 (1.24-4.64) | 1.02 (0.89-1.17) | 2.23 (1.15-4.35) |
| - 4 to <6 | 2641 | 715/30 | 1.29 (1.12-1.49) | 2.04 (1.00-4.16) | 1.08 (0.93-1.25) | 1.69 (0.82-3.47) |
| - ≥6 | 1066 | 330/22 | 1.46 (1.23-1.73) | 5.22 (2.58-10.54) | 1.07 (0.89-1.28) | 3.43 (1.65-7.12) |
| **Physical activity level**  Females |  |  |  |  |  |  |
| - Highest | 4167 | 1099/97 | 1 | 1 | 1 | 1 |
| - Moderate | 6717 | 2124/179 | 1.30 (1.19-1.43) | 1.11 (0.87-1.42) | 1.27 (1.16-1.39) | 1.03 (0.80-1.32) |
| - Light | 3256 | 1209/102 | 1.62 (1.46-1.79) | 1.57 (1.20-2.04) | 1.49 (1.34-1.66) | 1.24 (0.95-1.62) |
| - Lowest | 1661 | 759/75 | 2.45 (2.18-2.76) | 3.06 (2.33-4.01) | 2.14 (1.90-2.42) | 2.05 (1.54-2.71) |
| Males |  |  |  |  |  |  |
| - Highest | 3474 | 617/25 | 1 | 1 | 1 | 1 |
| - Moderate | 3824 | 949/35 | 1.41 (1.25-1.59) | 1.24 (0.77-1.99) | 1.38 (1.22-1.56) | 1.14 (0.71-1.84) |
| - Light | 2100 | 701/31 | 2.24 (1.96-2.55) | 2.11 (1.30-3.44) | 2.12 (1.85-2.43) | 1.62 (0.98-2.69) |
| - Lowest | 1226 | 419/30 | 2.32 (2.01-2.68) | 3.12 (1.92-5.06) | 2.17 (1.87-2.53) | 2.08 (1.24-3.49) |

*SI=Suicidal ideation, SA=Suicide attempt, RRR=Relative risk ratio.

**^#^**Weighted estimates

^†^Adjusted for parental educational level, parental income level, parental occupational status, family living situation, parental history of mental

disorder, adolescent’s smoking experience, adolescent’s alcohol experience, and the analysis for screen time is adjusted for physical activity

levels and the analysis for physical activity levels is adjusted for screen time.

**eTable 9:** Relative risk ratio of suicidal levels according to suicidal outcomes from the last 12 months only for females and males, respectively.

|  |  |  | Unadjusted^#^ | |
| --- | --- | --- | --- | --- |
|  | **N** | **SI/SA**  **n** | **Suicidal ideation**  **RRR (95% CI)** | **Suicide attempt**  RRR (95% CI) |
| **Screen time (hours per day)** Females |  |  |  |  |
| - <2 | 4614 | 937/26 | 1 | 1 |
| - 2 to <4 | 8502 | 1916/64 | 1.13 (1.03-1.24) | 1.70 (1.08-2.68) |
| - 4 to <6 | 2999 | 771/26 | 1.36 (1.22-1.52) | 2.01 (1.19-3.37) |
| - ≥6 | 986 | 289/14 | 1.59 (1.37-1.85) | 4.09 (2.32-7.20) |
| Males |  |  |  |  |
| - <2 | 1976 | 308/5 | 1 | 1 |
| - 2 to <4 | 5421 | 931/17 | 1.10 (0.95-1.28) | 0.76 (0.31-1.86) |
| - 4 to <6 | 2897 | 556/13 | 1.27 (1.09-1.49) | 1.24 (0.50-3.10) |
| - ≥6 | 1218 | 233/6 | 1.20 (0.99-1.45) | 2.07 (0.80-5.35) |
| **Physical activity level**  Females |  |  |  |  |
| - Highest | 4427 | 834/34 | 1 | 1 |
| - Moderate | 7216 | 1575/44 | 1.21 (1.09-1.33) | 0.70 (0.47-1.06) |
| - Light | 3592 | 936/30 | 1.50 (1.35-1.68) | 1.07 (0.69-1.65) |
| - Lowest | 1866 | 568/22 | 1.89 (1.68-2.13) | 1.71 (1.09-2.68) |
| Males |  |  |  |  |
| - Highest | 3671 | 459/9 | 1 | 1 |
| - Moderate | 4141 | 704/11 | 1.27 (1.11-1.45) | 0.82 (0.36-1.86) |
| - Light | 2354 | 558/8 | 2.02 (1.76-2.33) | 0.96 (0.38-2.42) |
| - Lowest | 1346 | 307/13 | 1.96 (1.68-2.29) | 3.17 (1.50-6.69) |

SI=Suicidal ideation, SA=Suicide attempt, RRR=Relative risk ratio.

**^#^**Weighted estimates

**eTable 10:** Relative risk ratio of suicidal levels according to sport participation and leisure

time physical activity among the weighted population of females and males, respectively.

|  |  |  | Adjusted^#†^ | |
| --- | --- | --- | --- | --- |
|  | **N** | **SI/SA**  **n** | **Suicidal ideation**  **RRR (95% CI)** | **Suicide attempt**  **RRR (95% CI)** |
| **Sport participation**  Females |  |  |  |  |
| - Yes | 14,716 | 4814/460 | 1 | 1 |
| - No | 2385 | 1074/141 | 1.58 (1.45-1.73) | 1.89 (1.58-2.26) |
| Males |  |  |  |  |
| - Yes | 9766 | 2493/119 | 1 | 1 |
| - No | 1746 | 606/54 | 1.44 (1.30-1.61) | 1.94 (1.42-2.63) |
| **Leisure time physical activity**  Females |  |  |  |  |
| - Very active | 4502 | 1254/125 | 1 | 1 |
| - Pretty active | 7585 | 2514/253 | 1.27 (1.16-1.38) | 1.13 (0.92-1.40) |
| - A little active | 4071 | 1655/179 | 1.66 (1.51-1.83) | 1.59 (1.27-1.98) |
| - Not active | 943 | 465/44 | 2.16 (1.86-2.52) | 1.74 (1.25-2.41) |
| Males |  |  |  |  |
| - Very active | 3727 | 707/37 | 1 | 1 |
| - Pretty active | 4429 | 1197/53 | 1.43 (1.27-1.60) | 1.08 (0.72-1.60) |
| - A little active | 2211 | 744/57 | 1.97 (1.73-2.24) | 2.27 (1.53-3.37) |
| - Not active | 1145 | 451/26 | 2.46 (2.11-2.87) | 1.47 (0.89-2.43) |

SI=Suicidal ideation, SA=Suicide attempt, RRR=Relative risk ratio.

**^#^**Weighted estimates

^†^Adjusted for parental educational level, parental income level, parental occupational status, family living

situation, parental history of mental disorder, adolescent’s smoking experience, and adolescent’s alcohol

experience.

**eTable 11:** Relative risk ratio of suicidal levels according to screen time and physical

activity levels also adjusted for child mental health variables

|  |  |  | Adjusted^†^ | |
| --- | --- | --- | --- | --- |
|  | N | SI/SA  n | Suicidal ideation  RRR (95% CI) | Suicide attempt  RRR (95% CI) |
| **Screen time (hours per day)**  Females |  |  |  |  |
| - <2 | 4614 | 1403/127 | 1 | 1 |
| - 2 to <4 | 8502 | 2899/281 | 1.1 (1.02-1.21) | 1.17 (0.95-1.45) |
| - 4 to <6 | 2999 | 1146/131 | 1.19 (1.07-1.32) | 1.41 (1.10-1.80) |
| - ≥6 | 986 | 440/62 | 1.39 (1.20-1.62) | 1.43 (1.05-1.96) |
| Males |  |  |  |  |
| - <2 | 1976 | 465/14 | 1 | 1 |
| - 2 to <4 | 5421 | 1388/79 | 1.00 (0.88-1.14) | 2.49 (1.36-4.57) |
| - 4 to <6 | 2897 | 843/46 | 1.08 (0.93-1.24) | 2.06 (1.09-3.91) |
| - ≥6 | 1218 | 403/34 | 1.06 (0.89-1.26) | 3.44 (1.79-6.61) |
| **Physical activity level**  Females |  |  |  |  |
| - Highest | 4427 | 1224/119 | 1 | 1 |
| - Moderate | 7216 | 2380/234 | 1.27 (1.16-1.39) | 1.18 (0.94-1.47) |
| - Light | 3592 | 1404/138 | 1.41 (1.27-1.56) | 1.22 (0.95-1.56) |
| - Lowest | 1866 | 880/110 | 1.98 (1.76-2.23) | 1.91 (1.48-2.47) |
| Males |  |  |  |  |
| - Highest | 3671 | 692/32 | 1 | 1 |
| - Moderate | 4141 | 1097/51 | 1.36 (1.21-1.53) | 1.11 (0.73-1.70) |
| - Light | 2354 | 834/48 | 1.97 (1.73-2.25) | 1.74 (1.13-2.70) |
| - Lowest | 1346 | 476/42 | 1.96 (1.69-2.27) | 2.41 (1.54-3.77) |

SI=Suicidal ideation, SA=Suicide attempt, RRR=Relative risk ratio.

**^#^**Weighted estimates

^†^Adjusted for parental educational level, parental income level, parental occupational status, family living

situation, parental history of mental disorder, adolescent’s smoking experience, adolescent’s alcohol

experience, SiC, depression symptoms, DSH, number of close friends, sleep, and adolescent’s history of mental disorder.

**
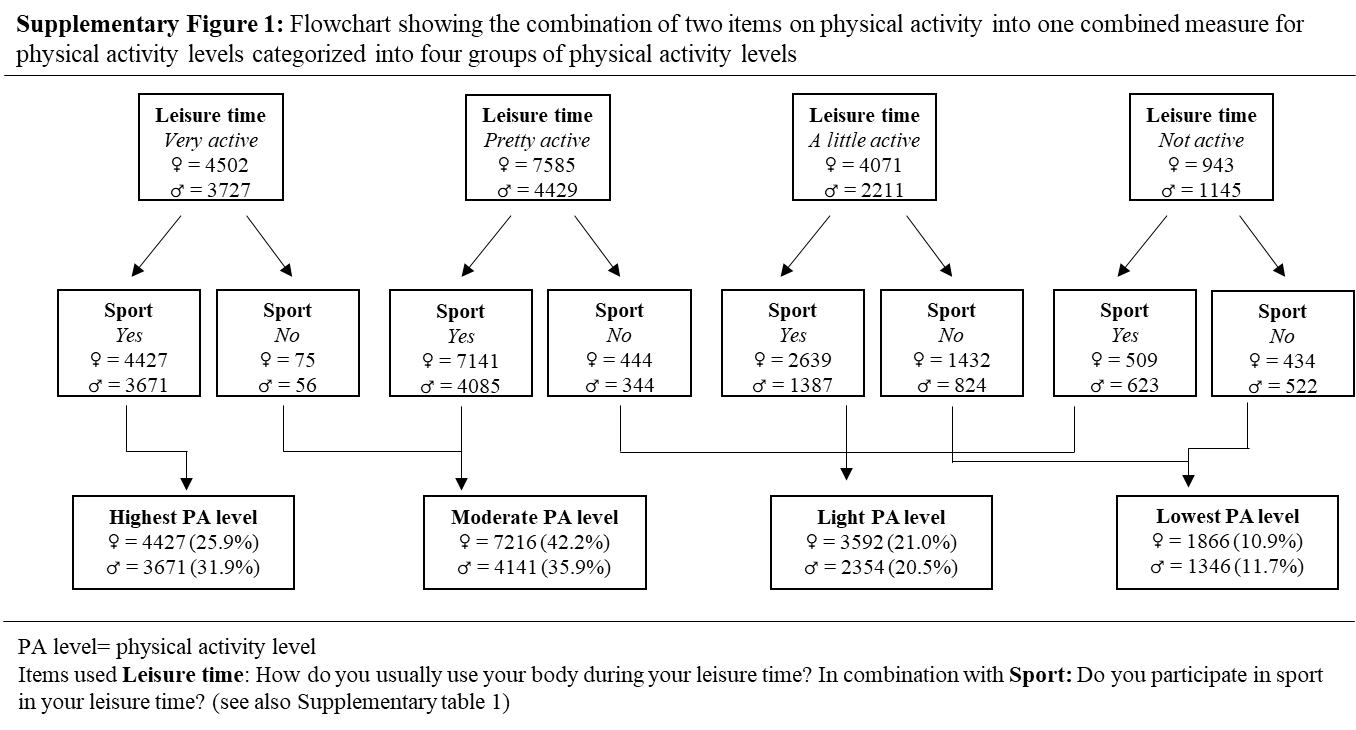
**
